# Supplementary material for: A preclinical model of THC edibles that produces high-dose cannabimimetic responses
Source: eLife. 2024 Jan 12;12:RP89867. doi: 10.7554/eLife.89867 (PMC10945583; doi:10.7554/eLife.89867)
Supplement: Figure 3—source data 1. [file elife-89867-fig3-data1.docx]

| **Brain (pmol/g)** | | | | | | |
| --- | --- | --- | --- | --- | --- | --- |
| **Compound** | **Collection time** | **Sex** | **Mean** | **SD** | **N** | **Significance** |
| THC | 1h | M | 557.2 | 263.2 | 9 | ns |
|  |  | F | 397.7 | 123.8 | 7 |  |
|  | 2h | M | 661.8 | 261.2 | 5 | ns |
|  |  | F | 501.6 | 490.6 | 5 |  |
|  | 2.5h | M | 573.8 | 303.9 | 8 | ns |
|  |  | F | 289.4 | 126.9 | 8 |  |
|  | 26h | M | 26.6 | 15.3 | 6 | ns |
|  |  | F | 67.8 | 60.6 | 6 |  |
| 11-OH-THC | 1h | M | 367.2 | 207.5 | 9 | ns |
|  |  | F | 356.2 | 75.5 | 7 |  |
|  | 2h | M | 406.7 | 124.7 | 5 | ns |
|  |  | F | 610.6 | 678.4 | 4 |  |
|  | 2.5h | M | 378.3 | 253.4 | 8 | ns |
|  |  | F | 268.7 | 177.3 | 8 |  |
|  | 26h | M | 10.6 | 16.8 | 6 | ns |
|  |  | F | 69.4 | 53.3 | 6 |  |
| COOH-THC | 1h | M | 200.8 | 227.8 | 6 | ns |
|  |  | F | 188.0 | 226.4 | 7 |  |
|  | 2h | M | 309.9 | 313.7 | 4 | ns |
|  |  | F | 371.4 | 354.4 | 4 |  |
|  | 2.5h | M | 190.0 | 182.1 | 8 | ns |
|  |  | F | 224.3 | 258.6 | 8 |  |
|  | 26h | M | 0.0 | 0.0 | 6 | ns |
|  |  | F | 31.1 | 52.9 | 6 |  |
