# Supplementary material for: A preclinical model of THC edibles that produces high-dose cannabimimetic responses
Source: eLife. 2024 Jan 12;12:RP89867. doi: 10.7554/eLife.89867 (PMC10945583; doi:10.7554/eLife.89867)
Supplement: Figure 3—source data 2. [file elife-89867-fig3-data2.docx]

| **Plasma (pmol/ml)** | | | | | | |
| --- | --- | --- | --- | --- | --- | --- |
| **Compound** | **Collection time** | **Sex** | **Mean** | **SD** | **N** | **Significance** |
| THC | 1 h | M | 478.5 | 276.7 | 8 | ns |
|  |  | F | 388.7 | 240.4 | 7 |  |
|  | 2 h | M | 235.4 | 122.3 | 5 | ns |
|  |  | F | 51.5 | 36.5 | 6 |  |
|  | 2.5 h | M | 115.8 | 75.8 | 8 | ns |
|  |  | F | 70.1 | 54.9 | 7 |  |
|  | 26 h | M | 3.3 | 6.2 | 7 | ns |
|  |  | F | 7.6 | 9.6 | 6 |  |
| 11-OH-THC | 1h | M | 62.4 | 25.9 | 7 | ns |
|  |  | F | 66.5 | 28.4 | 6 |  |
|  | 2h | M | 53.9 | 23.3 | 5 | ns |
|  |  | F | 36.0 | 6.2 | 3 |  |
|  | 2.5h | M | 58.9 | 30.3 | 7 | ns |
|  |  | F | 47.2 | 27.4 | 5 |  |
|  | 26h | M | 0.0 | 0.0 | 6 | ns |
|  |  | F | 4.6 | 7.2 | 6 |  |
| COOH-THC | 1h | M | 167.3 | 79.4 | 8 | ns |
|  |  | F | 472.4 | 207.4 | 7 |  |
|  | 2h | M | 116.3 | 52.9 | 5 | ns |
|  |  | F | 179.3 | 217.6 | 7 |  |
|  | 2.5h | M | 226.4 | 172.4 | 8 | ns |
|  |  | F | 194.3 | 99.5 | 7 |  |
|  | 26h | M | 14.7 | 24.2 | 6 | ns |
|  |  | F | 90.0 | 79.6 | 6 |  |
